# Neuron loss and degeneration in the progression of TDP-43 in frontotemporal lobar degeneration

**DOI:** 10.1186/s40478-017-0471-3

**Published:** 2017-09-06

**Authors:** Ahmed Yousef, John L. Robinson, David J. Irwin, Matthew D. Byrne, Linda K. Kwong, Edward B. Lee, Yan Xu, Sharon X. Xie, Lior Rennert, EunRan Suh, Vivianna M. Van Deerlin, Murray Grossman, Virginia M.-Y. Lee, John Q. Trojanowski

**Affiliations:** 1 Center for Neurodegenerative Disease Research and Institute on Aging, University of Pennsylvania Perelman School of Medicine, Philadelphia, PA 19104 USA; 2 Department of Neurology, University of Pennsylvania Perelman School of Medicine, Philadelphia, PA 19104 USA; 3 Department of Biostatistics and Epidemiology, University of Pennsylvania Perelman School of Medicine, Philadelphia, PA 19104 USA

**Keywords:** TDP-43, *C9orf72*, *GRN*, Frontotemporal lobar degeneration, NeuN, Neurodegeneration

## Abstract

Frontotemporal lobar degeneration with TDP-43 inclusions (FTLD-TDP) is associated with the accumulation of pathological neuronal and glial intracytoplasmic inclusions as well as accompanying neuron loss. We explored if cortical neurons detected by NeuN decreased with increasing TDP-43 inclusion pathology in the postmortem brains of 63 patients with sporadic and familial FTLD-TDP. Semi-automated quantitative algorithms to quantify histology in tissue sections stained with antibodies specific for pathological or phosphorylated TDP-43 (pTDP-43) and NeuN were developed and validated in affected (cerebral cortex) and minimally affected (cerebellar cortex) brain regions of FTLD-TDP cases. Immunohistochemistry (IHC) for NeuN and other neuronal markers found numerous neurons lacking reactivity, suggesting NeuN may reflect neuron health rather than neuron loss in FTLD. We found three patterns of NeuN and pTDP-43 reactivity in our sample of cortical tissue representing three intracortical region-specific stages of FTLD-TDP progression: Group 1 showed low levels of pathological pTDP-43 and high levels NeuN, while Group 2 showed increased levels of pTDP-43, and Group 3 tissues were characterized by reduced staining for both pTDP-43 and NeuN. Comparison of non-C9orf72/GRN FTLD-TDP with cases linked to both *GRN* mutations and *C9orf72* expansions showed a significantly increased frequency of Group 3 histopathology in the latter cases, suggesting more advanced cortical disease. Hence, we propose that IHC profiles of pTDP-43 and NeuN reflect the burden of pTDP-43 and its deleterious effects on neuron health.

**Electronic supplementary material:**

The online version of this article (10.1186/s40478-017-0471-3) contains supplementary material, which is available to authorized users.

## Introduction

Frontotemporal lobar degeneration (FTLD) is the second most common cause of neurodegenerative dementia in patients younger than 65 [21, 50]. Several pathological subtypes of FTLD have been identified including FTLD with TDP-43 inclusions (FTLD-TDP), FTLD associated with tau-positive inclusions (FTLD-Tau) or FUS-positive inclusions (FTLD-FUS) [18, 42, 46]. About 40% of FTLD patients have a family history of a neurodegenerative disease, and the most common genetic causes of FTLD include *C9orf72* expansions and mutations in the granulin precursor gene (*GRN*) [4, 11, 21]. Mutations in *C9orf72* are typified by a hexanucleotide repeat expansion of GGGGCC in the first intron of the promoter region of the gene [5, 11, 14, 47]. Importantly, TDP-43 inclusions are the hallmark brain lesions in the majority of familial FTLD cases. While TDP-43 inclusions accumulate mainly in the cerebrum, aggregated *C9orf72* dipeptide repeat peptides accumulate as TDP-43-negative but p62-positive neuronal cytoplasmic inclusions (NCI) in the cerebrum and cerebellum, but they rarely co-localize with TDP-43 inclusions [35, 49].

FTLD-TDP is a disorder characterized by diverse clinical, genetic, and pathological features [18, 42]. Macroscopic examination of the postmortem brains of patients diagnosed with clinical frontotemporal degeneration (FTD) generally reveals marked atrophy and neuronal loss, especially in the temporal and frontal lobes at end stage disease. In FTLD-TDP, normal nuclear TDP-43 is abnormally redistributed as insoluble phosphorylated TDP-43 (pTDP-43) into cytoplasmic inclusions accompanied by loss of nuclear TDP-43 in affected neurons and glia of the brain and spinal cord. Aggregations of TDP-43 appear as TDP-43-positive NCIs, dystrophic neurites (DN), and glial cytoplasmic inclusions (GCI) [42]. FTLD-TDP is further divided into five subtypes based on the distribution of the TDP-43 inclusions: subtype A consists of many NCI’s and DN’s in superficial cortical layers and is associated with *GRN* mutations; subtype B consists of moderate NCI’s and few DN’s throughout deep and superficial cortical layers and is associated with *C9orf72* mutations; subtype C consists of few NCI’s and many long DN’s in superficial cortical layers; subtype D consists of moderate numbers of intra-nuclear NCI’s in the deep and superficial cortical layers and is associated with pathogenic mutations in the gene encoding the valosin-containing protein (*VCP*); and the recently described subtype E variant of FTLD-TDP consists of granulofilamentous NCI and dot-like neuropil aggregates throughout all cortical layers without any known associated mutations [24, 25, 28, 31]. Subtypes A, B, and E also contain many TDP-43 positive GCI [24, 26]. As recently described, some cases have features of both subtypes A and B [28]. Furthermore, FTLD can present with overlapping clinical amyotrophic lateral sclerosis (ALS), a motor neuron disease typically marked by underlying TDP-43 pathology [9, 18]. FTLD that presents with symptoms of a motor neuron disease (e.g. muscle weakness and atrophy, loss of fine movements, dysphagia, and other motor difficulties) may be classified as FTLD-ALS [18, 57]. Moreover, FTD-TDP and ALS are both distinguished by regional distribution of TDP-43 pathology and may have similar genetic backgrounds [3, 9]. Notably, the *C9orf72* mutation is the most common cause of familial FTD, FTD-ALS, and ALS [40].

In healthy neurons, nuclear TDP-43 co-localizes with NeuN, a neuron-specific protein in vertebrates involved in RNA splicing [41]. Intense NeuN staining is seen in healthy neurons while reduced staining is commonly thought to be indicative of neurodegeneration or the compromised health of neurons in the absence of neuron death [10, 26, 56]. Neurodegeneration in FTLD-TDP is marked by the accumulation of TDP-43 inclusions, the loss of nuclear TDP-43, and the degeneration of neurons. Traditionally, disease staging is described based on the cortical distribution of pathology and resulting neurodegeneration on a whole-brain level [8, 9]. However, an understanding of how a disease progresses in a single brain region can also provide meaningful insight on the deleterious effects of pathology, genetic differences in disease severity, and the heterogeneity of disease subtypes. The purpose of this study is to investigate if NeuN immunohistochemical staining decreases with increasing levels of cortical TDP-43 severity. We employ quantitative pathology to establish three intracortical region-specific stages in FTLD-TDP.

## Materials and methods

### Autopsy tissue

For all autopsy cases utilized, written informed consent was obtained from all patients using a protocol approved by the University of Pennsylvania Institutional Review Board in addition to post-mortem consent from next of kin. All postmortem brains were retrieved from the brain bank at the Center for Neurodegenerative Disease Research (CNDR) at the University of Pennsylvania [52]. Neuropathologic diagnoses were established according to consensus criteria by expert neuropathologists (EBL, JQT) using immunohistochemistry (IHC) with established monoclonal antibodies specific for pathogenic tau (monoclonal antibody PHF-1; a gift from Dr. Peter Davies), TDP-43 (monoclonal antibody phospho(409/410)); a gift from Drs. Manuela Neumann and Elisabeth Kremmer), α-synuclein (monoclonal antibody Syn303; generated in CNDR), as well as amyloid-β (monoclonal antibody NAB228; generated in CNDR), as described previously [3, 18, 32, 38, 39, 52].

### Genetic analysis

Genomic DNA was extracted from brain tissues using QIAamp DNA mini kit (Qiagen, Germantown, MD) following manufacturer recommendations. Mutations and variants in *GRN* were screened by Sanger sequencing of the entire coding sequences of *GRN* and/or by targeted next generation sequencing (NGS) on a neurodegenerative disease-focused panel, MiND-Seq (Multi Neurodegenerative Disease Sequencing panel), which includes genes associated with FTD such as *GRN*, *MAPT*, *VCP*, *CHMP2B*, and *SQSTM1* [52, 59]. Sanger sequencing data were analyzed with Mutation Surveyor software (SoftGenetics, State College, PA) and alignment of sequence reads and variant calling from NGS were assessed by SureCall software (Agilent, Santa Clara, CA). *C9orf72* hexanucleotide repeat expansion was tested with repeat-primed PCR and capillary electrophoresis as previously described [51]. The sizes of the PCR fragments were analyzed with GeneMapper software (Applied Biosystems, Foster City, CA).

### Cohort

A cohort with neuropathologically confirmed FTLD-TDP was selected from patients autopsied between 1987 and 2015 (Table 1). It consisted of a convenience sample of 63 FTLD-TDP patients in the CNDR brain bank, 42 of which had comorbid pathologies. Sequential sections of mid-frontal and superior temporal cortices from 38 brains were stained for NeuN and pTDP-43 inclusions for each case. For a more expanded analysis of the cerebral cortex and cerebellum, nine cortical regions (orbital frontal, mid-frontal, superior temporal, entorhinal, anterior cingulate, motor, sensory, angular, and visual cortices) and the cerebellum from 25 brains were sampled and stained for NeuN and pTDP-43 inclusions. In total, 276 cortical sections from these 63 patients were analyzed. We had missing tissue for the mid-frontal (*n* = 7), superior temporal (*n* = 6), entorhinal (*n* = 1), anterior cingulate (*n* = 1), motor (*n* = 2), sensory (*n* = 5), angular (*n* = 1), and visual (*n* = 2) cortices. *GRN* cases were excluded from the analysis of cerebellar neuron health to compare *C9orf72* cases and those without either the *C9orf72* or *GRN* mutations (non-C9/GRN).

**Table 1:** Cohort characteristics

*n*	63
FTLD Type (A, B, C, E)	17, 21, 20, 5
Age onset	62.1 (9.2)
Age Death	68.8 (10.2)
Sex (m, f)	32, 31
Brain Wt	1110.0 g (188.6)
*GRN* Cases	12
*C9orf72* Cases	17
Clinical Diagnosis	
AD	8
ALS	3
CBD	3
FTD-bvFTD	22
FTD-NOS	14
FTD-PPA	12
MID	1
PSP	1
Primary Neuropath Diagnosis	FTLD-TDP
Secondary Neuropath Diagnosis	
AD	9
AGD	5
ALS	1
HS	2
LBD	1
None	21
PART	24

All data are expressed as mean (standard deviation). Clinical diagnoses reflect a patient’s diagnosis at time of death. *AD* alzheimer’s disease, *AGD* argyrophilic grain disease, *ALS* amyotrophic lateral sclerosis, *CBD* corticobasal syndrome, *FTD-bvFTD* behavioral variant FTD; *FTD-NOS FTD* not otherwise specified, *FTD-PPA* primary progressive aphasia, *HS* hippocampal sclerosis, *LBD* lewy body dementia, *MID* multi-infarct dementia, *PART* primary age-related tauopathy, *PSP* progressive supranuclear palsy

Immunoreactivity for several antibodies specific for other markers was tested in these tissue samples to understand the relevance of our analyses to neuron health. These included antibodies specific for splicing-factor proline and glutamine rich (SFPQ) protein, HuC/HuD RNA binding proteins, neurofilament heavy chain (NEFH), and astrocytic glial fibrillary acidic protein (GFAP). From our cohort, we selected five random cerebral cortex tissue sections from each Group for this analysis, as well as four clinically and pathologically normal cerebral cortex tissue samples as normal controls (NC). Four randomly selected NC were also used for comparison to FTLD in the analysis of cerebellar neuron health.

### Immunohistochemistry and Immunofluorescence

Tissue sections were subjected to IHC and immunofluorescence (IF) using previously published protocols [17, 52]. Briefly, after deparaffinization in xylene and rehydration through a series of increasing ethanol concentrations, the tissue sections were subjected to IHC using an avidin-biotin complex detection method with biotinylated anti-mouse, anti-rabbit, or anti-rat secondary antibodies and 0.05% 3,3-diaminobenzidine peroxidase substrate (Sigma D5637) as the chromogen. Hematoxylin was utilized as the counterstain. The following primary antibodies were used: pTDP-43 inclusions (rat monoclonal antibody p409/410 at a concentration of 0.06 μg/mL from Dr. Manuela Neumann); Pan TDP-43 (mouse monoclonal antibody 5104 at a concentration of 0.51 μg/ml from CNDR); NeuN (EMD Millipore Mab377 mouse monoclonal antibody NeuN at a concentration of 1.33 μg/mL); HuC/HuD (ThermoFisher Scientific A21271 mouse monoclonal antibody HuC/HuD at a dilution of 1:500); SFPQ (Abcam ab38148 rabbit polyclonal SFPQ at concentration of 1 μg/mL); RMO-24.9 (specific for phosphorylated NEFH; mouse monoclonal antibody at dilution of 1:2000 from CNDR), and 2.2B10 (specific for GFAP; rat monoclonal antibody at dilution of 1:5000 from CNDR). For IF, the NeuN and pTDP-43 antibodies were used at double the concentration used during IHC. To ensure that the 409/410 antibody was optimized for sections with low pTDP-43 antigenicity, all regions with less than 5 counts/mm^2^ of pTDP-43 were re-stained at a higher concentration (1:150), and the most optimally stained tissue was included in our cohort. Digital images were obtained using a Lamina Multilabel slide scanner (Perkin Elmer; Waltham, MA) with a 40× objective. The images had a pixel resolution of 0.2 μm/pixel, camera resolution of 2560 × 2160, and a bit depth of 16.

### Semi-automated quantification algorithms and selection of regions of analysis

Halo digital image software v2.0.1061.3 (Indica Labs; Albuquerque, NM) was used to develop detection algorithms to quantify pTDP-43 positive inclusions and NeuN staining. Specifically, the “Area Quantification” v1.0 setting (NeuN) and “CytoNuclear” v1.4 setting (TDP-43 inclusions and NeuN) were used to quantify NeuN reactive nuclei and TDP-43 inclusions, respectively. Previous work has validated the utility of these tools in detecting IHC-stained human tissue [17]. For each antibody, stains of interest were distinguished by red, green, and blue optical density (OD) for color deconvolution to isolate chromagen signals from their counterstain. For the “Area Quantification” algorithm, the threshold for positive OD—representing positive NeuN signal—was determined by visual inspection and cross-validated by multiple investigators (AY, JLR). The “CytoNuclear” algorithms are designed to detect cytoplasmic or nuclear positivity in individual cells. For these algorithms, a protocol was developed to validate automatic counts produced by Halo. Morphological and size characteristics were manipulated to develop the algorithms of interest. Initial parameters were set using the “real-time tuning” function of Halo. In all tissue used for algorithm development, regions of interest included all available grey matter in cerebral cortex or granular layer tissue of the cerebellum and excluded areas of tissue folding or shredding using the “exclusions drawing” tool. The pTDP-43 inclusions algorithm quantifies NCI and DN in aggregate while excluding diffuse pTDP-43 threads. For all tissue sections analyzed, quantification results are reported as percent area occupied or pTDP-43 positive inclusions/mm^2^.

Algorithm verification was done using tissue from our cohort. When defining algorithm parameters, all tissue were chosen at random and observers were blind to diagnostic information pertaining to the case. One out of six of the tissue sections analyzed here was used to validate each algorithm. After a random number generator was used to select regions for validation of these “CytoNuclear” algorithms, the grey matter of each tissue section was annotated using the “pen” tool. Tiles of 300-1500 μm^2^ were then partitioned within the annotated region using the “tile portioning tool.” A random number generator selected which tiles to use for manual counts. Enough tiles were selected to represent at least 5% of the grey matter area of each tissue section. Manual counts of IHC positive profiles in selected tiles were aided by the “manual click counter” tool and followed by automatic counts that were completed by the algorithm. Visual inspection of all analyzed tissue was done to ensure the algorithms properly detected their targets. In the case of algorithm failure (<10% of all analyzed tissue), small adjustments in OD were made to detect positive staining.

### Protein preparation and Immunoblotting

Sequential biochemical fractionation of human brain tissue was performed for four cases from our cohort (three mid-frontal and one superior temporal tissue sections), as previously described [1, 42]. Briefly, 1.2 g of grey matter was sequentially extracted in buffers of increasing strength (5 mL/g of tissue). The first extraction was with 1% Triton X-100 in high-salt buffer (HS-TX; 10 mM Tris-HCl, pH 7.4, 0.5 M NaCl, 2 mM EDTA, 10% sucrose (w:v), 1% Triton X-100 (v:v), and 1 mM DTT) and included protease/phosphatase inhibitors. The tissue was then homogenized and centrifuged at 180,000 for 30 min at 4 °C prior to resuspension in HS-TX buffer with 20% sucrose to remove myelin from the pellet. This pellet was then homogenized in nuclease buffer (50 mM Tris-HCl, pH 8.0, 20 mM NaCl, 5 mM MgCl2; 1 mL/g tissue) and incubated with BitNuclease (500 U/ g tissue, Biotool Co, Houston, TX) for 30 min on ice. Following this, the pellet was extracted with HS buffer containing 2% sarkosyl at 3.5 mL/g of tissue. The pellet was then washed in PBS at 3 mL/g and re-suspended in PBS at 250 μL/g followed by sonication using a hand-held probe (QSonica, Newtown, CT). Immunoblotting was performed as previously described [16, 42]. The 2% sarkosyl extract was loaded by volume (10 μL from each case) and separated on a 10% Tris-glycine SDS-PAGE followed by a transfer onto a 0.45 μM nitrocellulose membrane. The membrane was then blocked with Odyssey blocking buffer (LI-COR Biotechnology, Lincoln, NE) and probed with the mouse monoclonal antibody NeuN (Mab377; 1 g/mL; EMD Millipore). Positive immunoreactive signal was visualized using the secondary antibody IRDye 680RD goat anti-mouse IgG (926-32,210, Li-Cor) with a Li-Cor Odyssey imaging system.

### Statistical analysis

**Fig. 1:**
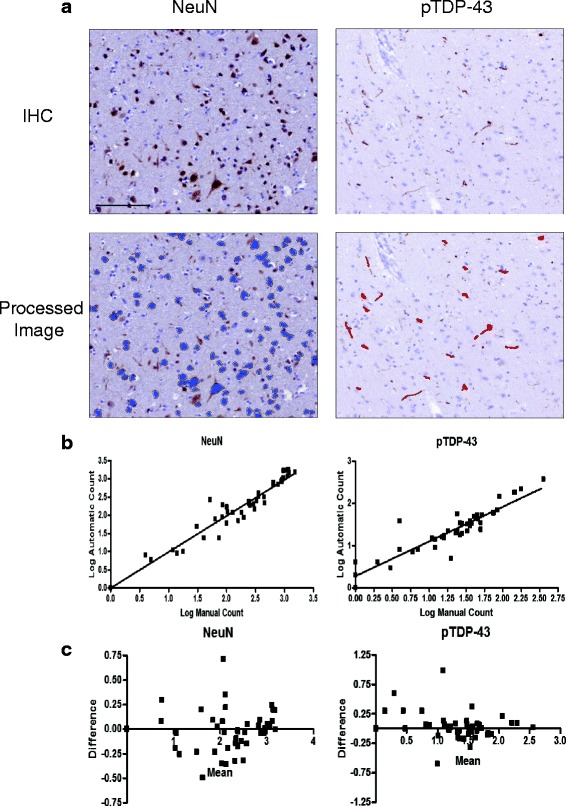
Algorithm development. Validation of the semi-automated quantification algorithms is shown through **a** representative images of the detection of NeuN and pTDP-43 by IHC (blue and red denote algorithm recognition in the processed image), **b** log-transformed regressions comparing automatic counts to manual counts (NeuN ICC = 0.959; pTDP-43 ICC = 0.913), and **c** Bland-Altman plots of the log-transformed data to test mean bias (NeuN = −0.019; pTDP-43 = 0.055) and 95% limit of agreement (NeuN = −0.440 to 0.402; pTDP-43 = −0.435 to 0.544) between automatic and manual counts. Bar = 100 μL

**Fig. 2:**
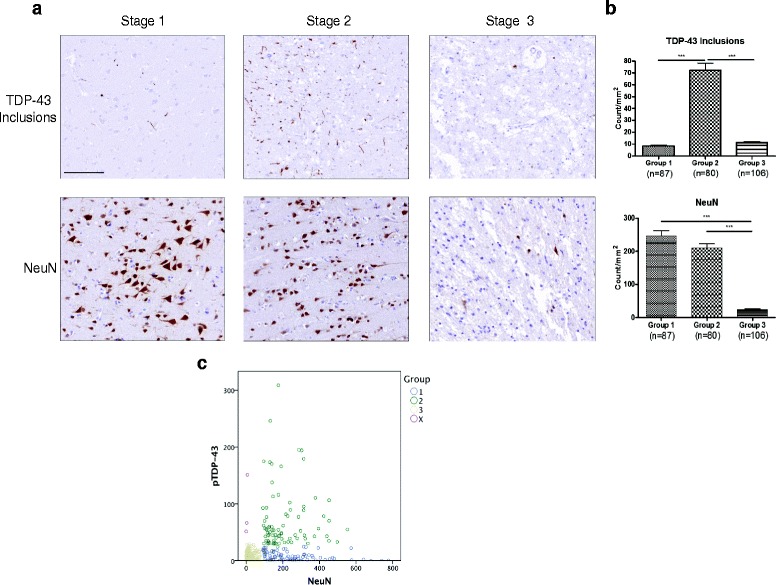
FTLD-TDP cerebral cortex is marked by three tissue grouping denoted by differences in the burden of pTDP-43 inclusions and NeuN positive neuronal nuclei stained by IHC. Progression of FTLD-TDP implicates three Groups of the state of cerebral cortex tissues shown by **a** IHC in representative images. In Group 1, neuron health is maintained as pathologic pTDP-43 begins to aggregate. Group 2 indicates the peak aggregation of pTDP-43 inclusions. In Group 3, pTDP-43 inclusions and healthy neurons simultaneously lose their immunoreactivity or disappear. Based on our data, we infer that the three Groups represent the sequential stages in the progression of FTDL-TDP in the cerebral cortex regions studies here. Evidence of neurodegeneration increases from Group 1 to Group 3 which is end stage FTLD-TDP disease **b** Quantification of the tissue in each Group indicates increase in pTDP-43 inclusions in Group 2 (*p* < 0.001) compared to Group 1, as well as a loss of pTDP-43 pathology in Group 3 compared to Group 2 (*p* < 0.001). NeuN quantification notes a loss of antigenicity in Group 3 when compared to Group 1 (*p* < 0.001) and Group 2 (*p* < 0.001). A Kruskal-Wallis test (*p* < 0.0001 and *p* < 0.0001, respectively) followed by Dunn’s test are used to assess significance for both pTDP-43 inclusions and NeuN nuclear staining. The categorization of each tissue section is noted in the scatter plot in (**c**). Axes are expressed in counts/mm^2^. For Group 1, *n* = 87; Group 2, *n* = 80; and Group 3, *n* = 106. Bar = 100 μL

Our primary interest here was to evaluate region-specific associations between NeuN and the pathologic burden of pTDP-43. ANOVA and Kruskall-Wallis tests using GraphPad software were applied to compare groups for normally and non-normally distributed data, respectively, followed by Tukey’s HSD test or Dunn’s test if significant [45]. Normal distributions were assessed by the Shapiro-Wilk test and plots. Additionally, intraclass correlation coefficients (ICC) and the Bland-Altman method were used to assess the reliability of quantification algorithms (Fig. 1b). To account for correlations among repeated measures (multiple regions sampled from a single brain), generalized estimating equations (GEE) using a proportional odds model were used to estimate odd’s ratios (OR) in the analyses of the effect of region and mutation on Group assignment in all tissue, as well as the bvFTD subset analysis; for the superior temporal cortex subset analysis, Fisher’s exact test is used [62]. Fisher’s exact test is also used in the analysis of FTLD-TDP subtypes and comorbidities. For each test, statistical significance is set to <0.05. SPSS Statistics Version 24 was used to produce ICC values, Fischer’s exact test, and to define the Groups indicated in Fig. 2c. GEE analysis was conducted using the statistical software package SAS version 9.4 (SAS Institute Inc., Cary, North Carolina). In creating these Groups, the mean pTDP-43 density count (~29 counts/mm^2^) of all tissue quantified was used as a cutoff for high TDP-43 pathology. The cutoff for high NeuN (~90 counts/mm^2^) was defined by visual inspection of clustering and validated by their “silhouette measure of cohesion and separation” (*S*_*i*_) which generated 0.389 as the mean *S*_*i*_ value, representing a moderately cohesive cluster [2]. The Gower similarity coefficient was used to measure dissimilarity in determining a mean silhouette coefficient. We validated our Grouping by performing an unbiased clustering analysis using Ward’s method. Ward’s method reported that three clusters are valid, accounting for 75.8% of the variance. The kappa statistic between the three clusters defined by Ward’s method and our three groups was 0.565. Group X, an outlier group consisting of 3 sections, is excluded from our analyses. When data are expressed in graphs, the midline indicates the mean and the error bars represent the standard error.

## Results

### Semi-automated quantitative algorithm development

Tissue sections from our cohort were stained for NeuN and pTDP-43 inclusions to develop the counting algorithms used in this study, and they serve as a relative index of NeuN level and TDP-43 pathology (Fig. 1a). Log-transformed manual and semi-automatic counts were compared to assess the validity of the algorithms **(**Fig. 1b **)**. The correlation (ICC) between NeuN manual counts and automatic counts was 0.959. For pTDP-43 inclusions the ICC was 0.913. Furthermore, a Bland-Altman method was used to test agreement between the algorithm derived data and the manual counts by determining median bias and limits of agreement **(**Fig. 1c **)**. For both algorithms, the majority of the measurements were within limits of agreement and the bias was quite small (NeuN = −0.019; pTDP-43 inclusions = 0.055). Therefore, algorithm counts align well with those done manually. To ensure that variations in the sampled area of each tissue section did not influence the Groups that follow, we compared pTDP-43 and NeuN counts/mm^2^ to the area of analysis of each tissue section in our cohort using linear regression and found no linear correlation (R^2^ = 0.0269 and R^2^ = 0.0297, respectively).

### Three groups of pTDP-43 and NeuN positive profiles are detected in FLTD-TDP tissue

Counts for pTDP-43 inclusions and NeuN were obtained for our cohort’s cerebral cortex tissue (Table 2). The cohort consisted of 63 cases, 17 with the *C9orf72* expansions, 12 with *GRN* mutations and 34 non-C9/GRN cases. The cohort comprised 31 females and 32 males with a mean age at death of 68.8 years. Additionally, the mean brain weight was 1110.0 g. The pTDP-43 inclusions ranged from 0 to 308.8 counts/mm^2^, with a mean 28.9 counts/mm^2^. The range for NeuN staining was 0 counts/mm^2^ – 777.9 counts/mm^2^, with mean of 148.6 counts/mm^2^. Qualitatively, when a scatter plot of pTDP-43 pathology versus NeuN counts was generated and the mean pTDP-43 value was defined as a cutoff, three large Groups of subjects were observed **(**Fig. 2a **)**.

**Table 2:** Pathology and NeuN data

	All		Non-C9/GRN		*C9orf72*		*GRN*	
	pTDP-43	NeuN	pTDP-43	NeuN	pTDP-43	NeuN	pTDP-43	NeuN
Range	0-308.8	0-777.9	0.3-195.1	0-777.9	0-194.2	0.1-740.2	0.3-308.8	0.6-640.7
Median	14.0	118.6	16.3	149.8	15.1	99.4	10.1	39.6
Mean	28.9	148.6	30.2	190.8	25.0	126.6	39.5	110.6
SD	41.6	145.7	38.4	155.1	31.1	130.7	72.4	145.9

All data is expressed as counts/mm^2^

We defined Groups 1-3 as described above based on NeuN nuclear staining and pTDP-43 inclusion densities (Fig. 2a-c **,** Additional file 1: Figure S1). Group 1 consisted of tissue sections with low pTDP-43 inclusions and high NeuN nuclear staining (*n* = 87); Group 2 tissue sections showed a high burden of pTDP-43 inclusions and high level of nuclear NeuN positivity (*n* = 80); and Group 3 had a low burden of pTDP-43 inclusions and a low level of NeuN positive neuronal nuclei (*n* = 106). However, a small Group of 3 sections showed low NeuN and high pTDP-43 inclusion levels.

### Validation of neurodegeneration in group 3

**Fig. 3:**
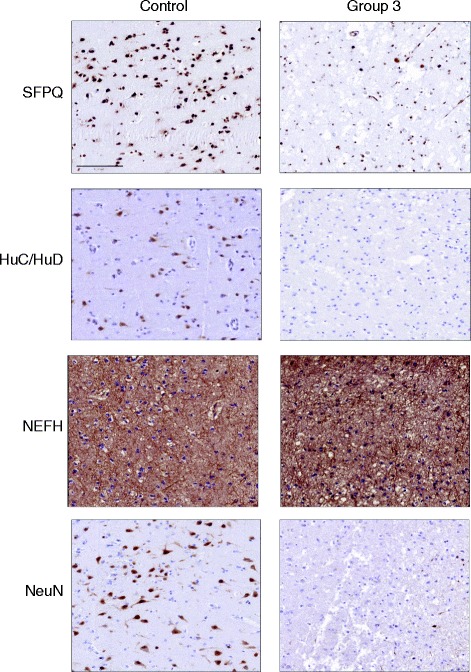
NeuN is a potential marker of neuronal health. This figure shows the loss of neuronal nuclear proteins in end stage FTLD-TDP (Group 3) compared to normal control cerebral cortex. This includes SFPQ, which is required for pre-mRNA splicing, and it diminishes from normal control to Group 3 cerebral cortex similar to the reduction of NeuN by IHC NeuN. HuC/HuD follows a similar staining pattern in control versus Group 3 cerebral cortex. A NEFH antibody is used to demonstrate axon integrity in the grey matter in control and Group 3 cerebral cortex. Bar = 100 μL

Notably, tissue in Group 3 exhibited many NeuN-negative neurons. To verify the loss of NeuN protein in Group 3, we randomly selected one case from Group 1, one case from Group 2, and two cases from Group 3 to sequentially extract and perform Western blot analysis on. A western blot of the 2% sarkosyl extract is shown in Additional file 1: Figure. S2a indicates significant reduction of NeuN in the Group 3 tissue (*n* = 2) compared to Groups 1 (*n* = 1) and 2 (*n* = 1). As further confirmation, IHC was performed with additional neuronal markers (Fig. 3). Group 1 and Group 3 tissue sections stained with HuC/HuD, a RNA binding protein, showed a similar pattern of staining as NeuN; SFPQ protein, an essential pre-mRNA splicing protein, showed low density of staining in Group 3 [61]. Therefore, by multiple measures, IHC detection of neurons is compromised in Group 3 even if neurons remain present. Conversely, other markers stain the tissue in Group 3 well. For instance, NEFH staining was still detected while astrocytosis was increased in Group 3 as reflected by GFAP IHC (Additional file 1: Figure S3**)**. Additionally, we find that all tissue maintain Pan TDP-43 level, regardless of Group assignment (Additional file 1: Figure S4).

### Groups 1-3 appear to recapitulate the distribution of pathological pTDP-43 in FTLD-TDP patients

In bvFTD, due to underlying pTDP-43 pathology (bvFTLD-TDP), it was found that pTDP-43 inclusion deposits generally aggregate anteriorly to posteriorly in the CNS [8]. 31 patients from our cohort were previously reported in Brettschneider, et al. [8]. In our study, we hypothesize that anterior brain regions would have a higher Group number (i.e. more advanced neurodegeneration) than posterior regions based on this previous model of four Phases of progression of TDP-43 pathology within the CNS [8]. We tested if this pattern was consistent with our findings by combining IHC data from our cohort into the these four Phases: Phase I consisted of the orbital frontal cortex (*n* = 24); Phase II consisted of the mid-frontal, anterior cingulate, entorhinal and superior temporal cortices (*n* = 161); Phase III consisted of the motor, sensory, and angular cortices (*n* = 65); and Phase IV consisted of the visual cortex (*n* = 23) (Table 3) as described by Brettschneider et al. [8]. Indeed, Phase assignment was associated with Group number (*p* = 0.0004). With an OR of 0.33 (95% CI 0.18-0.60), tissue in Phase I are 67% less likely than tissue in Phases III or IV to be in Groups 1 or 2 than Group 3. Likewise, tissue in Phases III or IV are 3.64 times more likely than tissue in Phase II to be in Groups 1 or 2 than 3 (OR = 3.64; 95% CI 2.14-6.18). There was no significant difference in Group number between Phases I and II (*p* = 0.6005).

**Table 3:** Grouping of cerebral cortex tissue sections indicates distinct regional distribution and genetic heterogeneity

Measure	Estimate	Standard error	Chi-squared with DF = 1 (*P*-Value)	OR (95% CI)	Chi-squared (DF) (*P*-Value)
Phase					
I^a^	−1.112	0.307	13.10 (0.0003)	0.33 (0.18-0.60)	15.72 (2) (0.0004)
II^b^	−0.180	0.344	0.27 (0.6005)	0.84(0.43-1.64)	
III/IV^c^	1.292	0.270	22.84 (<0.0001)	3.64 (2.14-6.18)	
Mutation					
Non-C9/GRN^d^	1.038	0.448	5.37 (0.0205)	2.82 (1.17-6.80)	7.80 (2) (0.0203)
*C9orf72* ^*e*^	−0.996	0.390	6.52 (0.0107)	0.37 (0.17-0.79)	
*GRN* ^f^	−0.043	0.465	0.01 (0.9269)	0.95 (0.39-2.38)	

^a^Phase III/IV is reference group

^b^I is reference group

^c^II is reference group

^d^ *GRN* is reference group

^e^Non-C9/GRN is reference group

^f^ *C9orf72* is the reference group

GEE using a proportional odds model for Group 1-2 based on 273 observations. I- orbital frontal cortex; II- mid-frontal, anterior cingulate, entorhinal and superior temporal cortices; III- motor, sensory, and angular cortices; IV- visual cortex. Regions in III and IV are combined due to small number of observations in each region

To control for clinical phenotypic variation in TDP-43 regional pathology, we performed a subset analysis in 22 patients with bvFTD as well as 41 non-bvFTD patients and found a similar pattern (Additional file 1: Table S1, S2).

### Groups 1-3 distinguish *C9orf72* and *GRN* FTLD-TDP

*C9orf72* expansions and *GRN* mutations have been shown to have regional patterns of disease within the CNS that differ from sporadic FTLD-TDP [7, 19, 29, 30, 33, 58]. *C9orf72* expansions have also been shown to cause decreased cognition in FTLD, suggesting a more aggressive disease course [19]. Therefore, if the Groups 1-3 described here have relevance to disease progression, the overall burden in non-C9/GRN cases would be expected to differ from those cases with pathogenic mutations in *C9orf72* and *GRN*. To test this, we compared Group number in tissue sections from *C9orf72* expansion cases (*n* = 133), *GRN* mutation cases (*n* = 36), and non-C9/GRN cases (*n* = 104) using a GEE analysis, and found a significant difference (*p* = 0.0203) (Table 3). Non-C9/GRN tissue are 2.82 (OR = 2.82; 95% CI 1.17-6.80) times more likely than tissue with the *GRN* mutation to be in Group 1 or 2 than 3. Similarly, *C9orf72* tissue are 63% (OR = 0.37; 95% CI 0.17-0.79) less likely to be in Groups 1 or 2 than 3 compared to non-C9/GRN tissue. No significant difference was observed between *C9orf72* and *GRN* tissue (*p* = 0.9269). To account for clinical phenotypic differences and region-specific associations, we performed sub-analyses of both bvFTLD-TDP patients and non-bvFTLD-TDP as well as all superior temporal cortex tissue—a region affected early in FTLD-TDP—and found similar results **(**Additional file 1: Table S1-S3).

### FTLD-TDP subtypes a and B cases show augmented disease

All three Groups are found in FTLD-TDP subtypes A-C, yet Group 3 is not seen in subtype E **(**Table 4 **)**. However, they were unevenly distributed (*p* < 0.0001). Specifically, subtype A was comprised predominately of Group 3 tissue with fewer Group 1 or 2 tissue. Subtype B showed a similar, although much less pronounced trend, while subtype C was evenly distributed across all three Groups and subtype E was purely in Groups 1 and 2. Furthermore, we find that comorbidity has no effect on Group categorization. Of the 42 cases with comorbidities, 28.5% of tissue are in Group 1, 34.6% are in Group 2, and 36.9% are in Group 3. A 2-sided Fisher’s exact test does not find a significantly different distribution of Groups between cases with and without comorbidities (*p* = 0.177).

**Table 4:** Distribution of groups in FTLD-TDP subtypes

	Group				
FTLD-TDP subtype	Count Total % Col % Row %	1	2	3	Total
A		10 3.7% 11.5% 25.0%	5 1.8% 6.3% 12.5%	25 9.2% 23.6% 62.5%	40 14.7%
B		46 16.8% 52.9% 31.5%	42 15.4% 52.5% 28.8%	58 21.2% 54.7% 39.7%	146 53.5%
C		22 8.1% 25.3% 33.3%	21 7.7% 26.2% 31.8%	23 8.4% 21.7% 34.8%	66 24.2%
E		9 3.3% 10.3% 42.9%	12 4.4% 15.0% 57.1%	0 0% 0% 0%	21 7.7%
Total		87 31.9%	80 29.3%	106 38.8%	273

A 2-sided Fisher’s exact test finds a significantly different distribution of Groups among the subtypes (*p* < 0.0001)

### Loss of cerebellar NeuN density in *C9orf72*

**Fig. 4:**
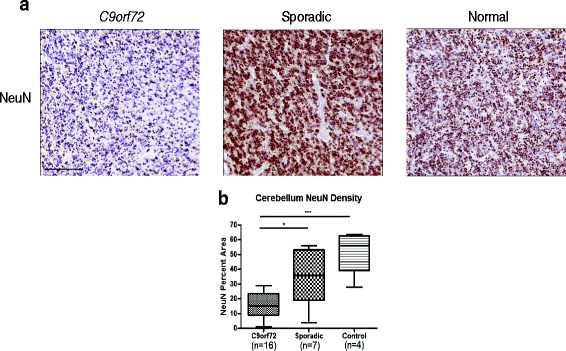
Cerebellum NeuN density decreases in *C9orf72*. **a** Representative images of NeuN IHC are shown for the granule cells of the cerebellum of FTLD-TDP cases due to the presence of a *C9orf72* expansion and non-C9/GRN FTLD-TDP, as well as a normal brain from a cognitively intact subject. **b** Quantification of NeuN percent area in this region reveals a marked decrease in antigenicity in *C9orf72* cases compared to non-C9/GRN (*p* < 0.05). A significant decrease was also observed with *C9orf72* cases compared to control (*p* < 0.001). ANOVA (*p* = 0.0002) followed by Tukey’s HSD test are used to assess significance. Bar = 100 μL

A unique signature of FTLD-TDP due to *C9orf72*expansions (C9FTLD-TDP) is the presence of dipeptide repeat inclusions in the cerebellum without the presence of TDP-43 pathology or overt neurodegeneration on postmortem examination, while neuroimaging studies find cerebellar atrophy of unclear significance [6, 40]. Cerebellar tissue sections from 23 non-C9/GRN and *C9orf72* FTLD-TDP cases as well as four NC subjects were stained with NeuN to determine if there was a loss of NeuN IHC density in the granule cells between cases with *C9orf72* expansion (*n* = 16) and those without (*n* = 7) (Fig. 4a, b). C9FTLD-TDP cerebellum (mean = 16.4%, STD = 8.4%) displayed decreased NeuN densities compared to both non-C9/GRN FTLD-TDP (mean = 34.4%, STD = 20.5%) and normal (mean = 50.9%, STD = 16.4%) cerebellum (*p* < 0.05, *p* < 0.001).

## Discussion

We investigated 63 FTLD-TDP cases and identified three Groups of histopathology that proceed from the aggregation of intracytoplasmic TDP-43 inclusions to progressive accumulation of TDP-43 inclusions followed by a reduction in these inclusions as well as reduced NeuN staining consistent with deteriorated neuron health. Indeed, in the cerebellar cortex, we find the granular layer neurons exhibit low NeuN staining in *C9orf72* cases. Additionally, *C9orf72* and *GRN* cases display a more advanced disease state, as defined by increased Group 3 frequency. Moreover, we found that Groups 1-3 can be used to model staging of the progression of pathology within an individual brain region and across brain regions.

Utilization of a NeuN antibody was essential to this study. NeuN was discovered to be a product of the Fox-3 gene in 2011, which functions as a splicing activator for exon N30 of NMHC II-B via the intronic UGCAUG element in neurons [20]. Variations in NeuN staining of neurons are observed in diseased CNS tissues, but a consistent pattern of NeuN corresponding to pathology is not well defined [10, 13]. In this study, we find that NeuN quantification is not an absolute measure of neuron loss but instead suggest that it reflects neuron health since many remaining neurons are NeuN negative in Group 3 IHC (Fig. 2), IF (Additional file 1: Figure S1), and western blot (Additional file 1: Figure S2a).

Importantly, we show a reduction in other neuronal markers in adjacent sections stained with SFPQ and HuC/HuD (Fig. 3). A 2015 study of a transgenic pig expressing mutant TDP-43 posits that TDP-43 interacts with SFPQ, a neuronal pre-mRNA splicing factor. This association of SFPQ with NeuN suggests that both proteins are disrupted in disease [55]. Furthermore, HuC/HuD proteins are neuronal RNA binding proteins known for their mRNA stabilizing property, and they are required for differentiation, maintenance and plasticity of neurons [44]. Moreover, previous work is consistent with the view that NeuN marks neuronal health, showing how NeuN expression in humans decreases under conditions such as perinatal death [23]. Likewise, *RBFOX3* (the gene encoding NeuN) knockout mice show decreased synapse activity and plasticity [56]. Therefore, we suggest that TDP-43’s cytoplasmic mislocalization decreases health by reducing NeuN expression in Group 3 as well as the expression of essential neuronal proteins. In future studies, caution must be maintained in interpreting a reduction of NeuN as a reflection of neuron loss. Indeed, NeuN levels should be validated with other neuronal markers because loss of NeuN antigenicity may be a consequence of other events such as cerebral ischemia, 17-Gy irradiation, and axotomy [37, 53, 60]. Additionally, agonal state, RNA quality, and comorbidities may have an effect on NeuN staining. In our cohort, we find that Pan TDP-43 levels are maintained in Groups 1-3, indicating that problems relating to IHC are not driving low NeuN staining.

Reduced NeuN staining also was observed in cerebellar granule cells of FTLD-TDP cases (Fig. 4) with *C9orf72* expansions. Interestingly, research has shown that the granule cells of the cerebellum are marked by pTDP-43-negative but p62-positive NCI [35]. This suggests that neuron loss in these cells of the cerebellum is not due to pathologic TDP-43. Instead, recent studies suggest that dipeptide repeat proteins, which are translated in *C9orf72* cases accumulate in the cerebellum and could play a role neuron loss in these cells, though a direct causative link has not been shown [30]. Notably, antemortem neuroimaging studies confirm cerebellar atrophy in *C9orf72* cases, and this is the first postmortem confirmation of this finding [6, 19, 34].

The toxicity of pathologic TDP-43 is well established. First, past work has shown that the burden of cytoplasmic pTDP-43 expression correlated with neurotoxicity in cultured cells [24]. In fact, overexpression of cytoplasmic pTDP-43 was generally toxic to neurons in animal models [24, 27, 54, 57]. Moreover, previous literature suggests that pathological TDP-43 is associated with loss of normal functionality [24, 27]. Together with human studies describing a progression of TDP-43 pathology as it spreads from one brain area to the next in both bvFTLD-TDP and ALS, pTDP-43 is clearly implicated as a toxic cause of FTLD-TDP [8, 9].

**Fig. 5:**
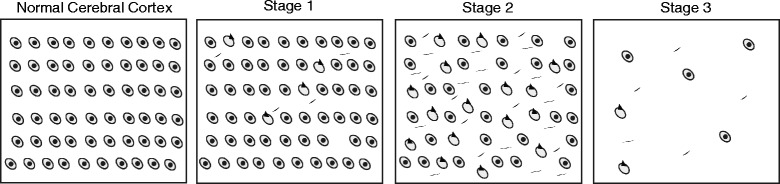
Proposed stages of intracortical region-specific decline in FTLD-TDP. This illustration defines three stages of regional decline in the cerebral cortex of FTLD-TDP that proceed from the aggregation of pTDP-43 inclusions to degeneration of tissue. NC is characterized by healthy neurons and a lack of pathology. In Stage 1, pathology begins to aggregate and neuronal health is maintained. Likewise, in Stage 2, neuronal health is maintained but an increase in pathological aggregates is observed. Lastly, Stage 3 is typified by a clearance of pathology, tissue degeneration, and depressed neuronal health. In this model, the presence of NeuN, SFPQ, and HuC/HuD proteins distinguishes healthy neurons

Our findings (Fig. 2) suggest that with increasing neurodegeneration there may be a reduction of pTDP-43 pathology, that Group 3 consists of end stage FTLD-TDP due to loss of NeuN, and that assignment to Groups 1-3 may reflect three Stages of progression of pathology in FTLD-TDP [43]. Thus, based on the data presented here, we propose the following model disease progression within a cortical region of FTLD-TDP cases (Fig. 5):

1. Stage 1- Very little pTDP-43 has been mislocalized to the cytoplasm and NeuN staining is similar to NC.
2. Stage 2- Pathologic pTDP-43 aggregates have accumulated into inclusions, but they have yet to show NeuN loss.
3. Stage 3- The toxicity of pathologic pTDP-43 is suggested by a significant increase in degeneration. As neuron health degrades and neurons die, we infer that pathologic pTDP-43 is cleared from the affected cerebral cortex [43]. Further, this stage marks a corresponding decrease of other nuclear neuronal proteins (e.g. SFPQ, HuC/HuD). Moreover, Groups 2 and 3 also show more evidence of gliosis **(**Additional file 1: Figure S3).

We also find 3 sections in Group X, which would be characterized by a high burden of pTDP-43 pathology and a low NeuN signal. The scarcity of tissue in this Group implies either that this Stage is transient or that neuron health and TDP-43 pathology degrade simultaneously.

A few factors may limit our findings. First, a patient’s clinical phenotype may influence the conclusions drawn from the Groups. However, we find that our conclusions on regional distribution of the Groups as well as genetic heterogeneity are replicated in bvFTD, the largest clinical phenotype in our cohort (Additional file 1: Table S1). Second, in this study, the entire grey matter is sampled in defining pathology and NeuN staining levels. Variable sectioning of tissue may over-represent specific cortical layers vulnerable to TDP-43 pathology (i.e. Layers II/III) and thereby misrepresent their Group assignment. Indeed, all randomness and bias cannot be excluded from the semi-automated quantification technique employed. Although semi-automated quantification enabled this study to be conducted in an efficient and timely manner, limitations of this technique include the time required to develop and validate detection algorithms, the technology needed for producing these algorithms, and exclusion of small or variable pathologies. In this study, we employ an algorithm to quantify pTDP-43 pathology and our quantification method is effective compared to manual counts **(**Fig. 1b, c **)**, but we excluded small diffuse TDP-43 threads from our analysis in order to improve the algorithm’s reliability. Since these pTDP-43 positive neuritic lesions are abundant in FTLD-TDP subtypes A and E, this strategy may have artificially decreased the frequency of Group 2 in these subtypes. Likewise, separate pTDP-43 quantification algorithms were not developed specifically for each subtype. Still, we find that Group 2 is well represented in both subtypes A and E (Table 4). Interestingly, we find an increased frequency of Group 3 in subtypes A and B. We also find no Group 3 in subtype E. We suspect that disease duration may be subtype specific since these three subtypes have a shorter disease duration (6.5, 6.0, and 2.6 years, respectively) compared to subtype C (9.0 years) (disease duration data was missing for one subtype A and one C case). Indeed, this distribution of Groups implies that subtypes A and B are subject to a more severe disease course and subtype E has a more rapid disease course. Furthermore, since 42 of the 63 cases analyzed in this study had comorbidities, it is possible that this may confound our conclusions. Still, these 42 cases had a fairly even distribution of Group Number (28.5% of tissue are in Group 1, 34.6% are in Group 2, and 36.9% are in Group 3), which matches well with the distribution through the entire cohort (Table 4).

Moreover, our data suggest more severe neurodegeneration in cases with the *C9orf72* and *GRN* mutations. Correspondingly, we found **(**Table 3 **)** that FTLD-TDP cases due to *C9orf72* and *GRN* mutations were more common in Group 3 than Groups 1 or 2. This implies that these cases have a more severe disease phenotype compared to non-C9/GRN FTLD, a hypothesis substantiated by previous studies which showed increased rates of decline in *C9orf72* cases and greater brain atrophy in *GRN* cases [7, 19, 29, 30, 33, 58]. Specifically, these studies have noted shorter survival, higher rates of decline in letter fluency, and increased cerebral and cerebellar atrophy in *C9orf72* [7, 19, 30, 33]. Cases with the *GRN* mutations displayed greater atrophy of the frontal, temporal, and parietal cortices [29, 58]. Yet, the finding of heightened clinical decline in *C9orf72* is not consistent in the literature. A study of Australian FTLD cases finds lessened atrophy and slower disease progression in *C9orf72* [12]. However, variation in these findings may be due to *C9orf72* methylation state, which has been shown to affect age of onset and neuron loss [15, 36, 48].

In addition to modeling intracortical region-specific staging of disease, we investigate the progression of pathology within an individual across brain regions (Fig. 2a). In bvFTLD-TDP, previous work has defined four Phases of TDP-43 distribution [8]. Other work has confirmed increased atrophy—and associated neuron loss—in anterior regions and less progressive atrophy in posterior regions of FTLD brains [22]. Here, we recapitulated the Phases in Brettschneider, et al. using our Groups (Table 3). Moreover, the increased frequency of Group 3 in anterior brain regions indicates accentuated neuron loss and atrophy compared to posterior brain regions. Therefore, we hypothesize that whole-brain staging of pTDP-43 driven neuropathologic decline in FTLD-TDP is marked by region-specific degeneration that is heightened in anterior brain regions and progresses sequentially to posterior brain regions. Ultimately, it is clear that a better understanding of the mechanisms involved in mislocalization of pTDP-43, its spread, and genetic heterogeneity could provide opportunities for treatment of TDP-43 proteinopathies.

## Additional file

Additional file 1: Figure S1. Three observed Groups denoted by differences in NeuN and TDP-43 inclusions are seen in IF. Representative images in IF confirms the pattern of staining seen through IHC. In Group 1, NeuN staining remains high with little aggregation of pathology. Pathology increases in Group 2, followed by a simultaneous decrease in both pathology and NeuN in Group 3. Cognitively normal controls maintain a high NeuN level and no pathology. Bar = 100 μL. **Figure S2.** Immunoblot confirms loss of NeuN protein in Group 3 tissue. After sequential extraction of 2% sarkosyl soluble fraction of mid-frontal and superior temporal cortex grey matter from Groups 1, 2, and 3 tissue, (a) they were immunoblotted with a NeuN antibody. Groups 1 and 2 maintained noticeably higher protein levels than Group 3. (b) The Ponceau S stain of the membrane is shown to demonstrate similar levels of protein transfer. **Figure S3.** Reactive gliosis increases with increasing Group number. As tissue progresses from Group 1 to Group 3, astrocytosis becomes increasingly evident in the grey matter. Representative images are shown. Bar = 500 μL. **Figure S4** Pan TDP-43 is maintained through Groups 1, 2 and 3. Validation of the semi-automated quantification algorithms is shown through (a) representative images of the detection of Pan TDP-43 by IHC (red denotes algorithm recognition in the processed image), (b) log-transformed regressions comparing automatic counts to manual counts (ICC = 0.998), and (c) Bland-Altman plots of the log-transformed data to test mean bias (−0.004) and 95% limit of agreement (−0.070 to 0.062) between automatic and manual counts. FTLD-TDP cerebral cortex is marked by three tissue grouping denoted by differences in the burden of pTDP-43 inclusions and NeuN positive neuronal nuclei stained by IHC. Available (slices sequential to those used for NeuN and pTDP-43 IHC) (*n* = 94) mid-frontal and superior temporal cortex tissue are selected to investigate staining of Pan TDP-43 in Groups 1-3. Although evidence of neurodegeneration increases from Group 1 to Group 3, we find that (d) Pan TDP-43 is maintained. (e) Quantification of the tissue in each Group also indicates this (Group 1, *n* = 33; Group 2, *n* = 14; Group 3, *n* = 47) (ANOVA, *p* = 0.1602). **Table S1.** Focused analyses of bvFTLD-TDP recapitulate spread of pathology and genetic differences. **Table S2.** Focused analyses of non-bvFTLD-TDP recapitulate spread of pathology and genetic differences. **Table S3.** Superior temporal cortex tissue recapitulates genetic differences in FTLD-TDP. (PDF 17529 kb)
