# Supplementary material for: Neuron loss and degeneration in the progression of TDP-43 in frontotemporal lobar degeneration
Source: Acta Neuropathol Commun. 2017 Sep 6;5:68. doi: 10.1186/s40478-017-0471-3 (PMC5586052; doi:10.1186/s40478-017-0471-3)
Supplement: Additional file 1: Figure S1. — Three observed Groups denoted by differences in NeuN and TDP-43 inclusions are seen in IF. Representative images in IF confirms the pattern of staining seen through IHC. In Group 1, NeuN staining remains high with little aggregation of pathology. Pathology increases in Group 2, followed by a simultaneous decrease in both pathology and NeuN in Group 3. Cognitively normal controls maintain a high NeuN level and no pathology. Bar = 100 μL. Figure S2. Immunoblot confirms loss of NeuN protein in Group 3 tissue. After sequential extraction of 2% sarkosyl soluble fraction of mid-frontal and superior temporal cortex grey matter from Groups 1, 2, and 3 tissue, (a) they were immunoblotted with a NeuN antibody. Groups 1 and 2 maintained noticeably higher protein levels than Group 3. (b) The Ponceau S stain of the membrane is shown to demonstrate similar levels of protein transfer. Figure S3. Reactive gliosis increases with increasing Group number. As tissue progresses from Group 1 to Group 3, astrocytosis becomes increasingly evident in the grey matter. Representative images are shown. Bar = 500 μL. Figure S4 Pan TDP-43 is maintained through Groups 1, 2 and 3. Validation of the semi-automated quantification algorithms is shown through (a) representative images of the detection of Pan TDP-43 by IHC (red denotes algorithm recognition in the processed image), (b) log-transformed regressions comparing automatic counts to manual counts (ICC = 0.998), and (c) Bland-Altman plots of the log-transformed data to test mean bias (−0.004) and 95% limit of agreement (−0.070 to 0.062) between automatic and manual counts. FTLD-TDP cerebral cortex is marked by three tissue grouping denoted by differences in the burden of pTDP-43 inclusions and NeuN positive neuronal nuclei stained by IHC. Available (slices sequential to those used for NeuN and pTDP-43 IHC) (n = 94) mid-frontal and superior temporal cortex tissue are selected to investigate staining of Pan TDP-43 in Groups 1-3. Although evidenc [file 40478_2017_471_MOESM1_ESM.pdf]

Electronic Supplementary Material

Neuron loss and degeneration in the progression of TDP-43 proteinopathy

Acta Neuropathologica

[1]Ahmed Yousef, [1]John L. Robinson, [1,2]David J. Irwin, [1]Matthew D. Byrne, [1]Linda K. Kwong, [1]Edward B. Lee, [1]Yan Xu, [3]Sharon X. Xie, [3]Lior Rennert, [1]EunRan Suh, [1]Vivianna M. Van Deerlin, [1, 2] Murray Grossman, [1]Virginia M.-Y. Lee, [1]John Q. Trojanowski

From

[1]Center for Neurodegenerative Disease Research and Institute on Aging, [2]Department of Neurology, [3]Department of Biostatistics and Epidemiology, University of Pennsylvania Perelman School of Medicine, Philadelphia, PA 19104, USA

*Correspondence to: trojanow@upenn.edu, 215-662-4474

Fig. S1

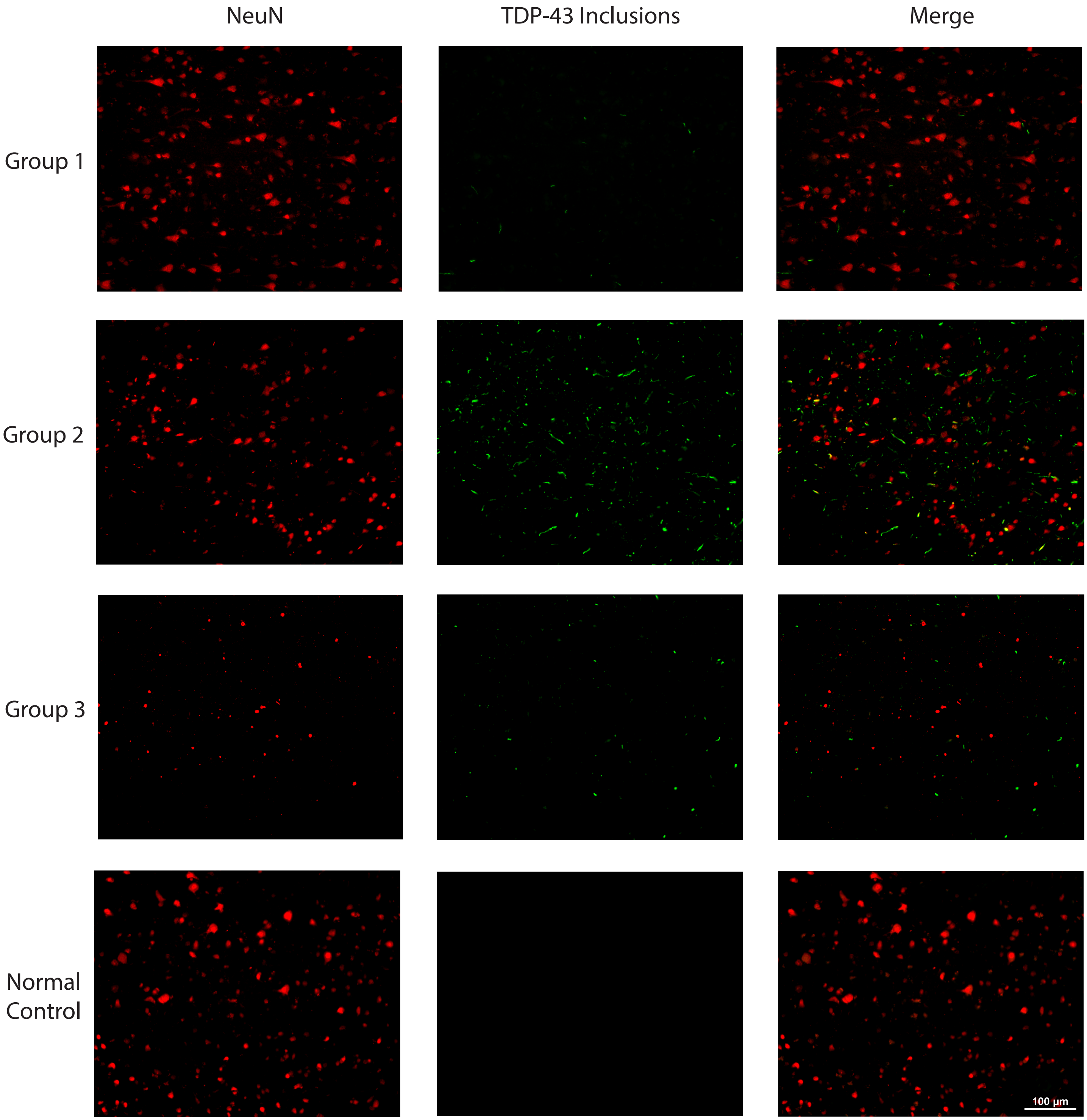
NeuN
TDP-43 Inclusions
Merge
Group 1
Group 2
Group 3
Normal
Control
100 µm

Fig. S2

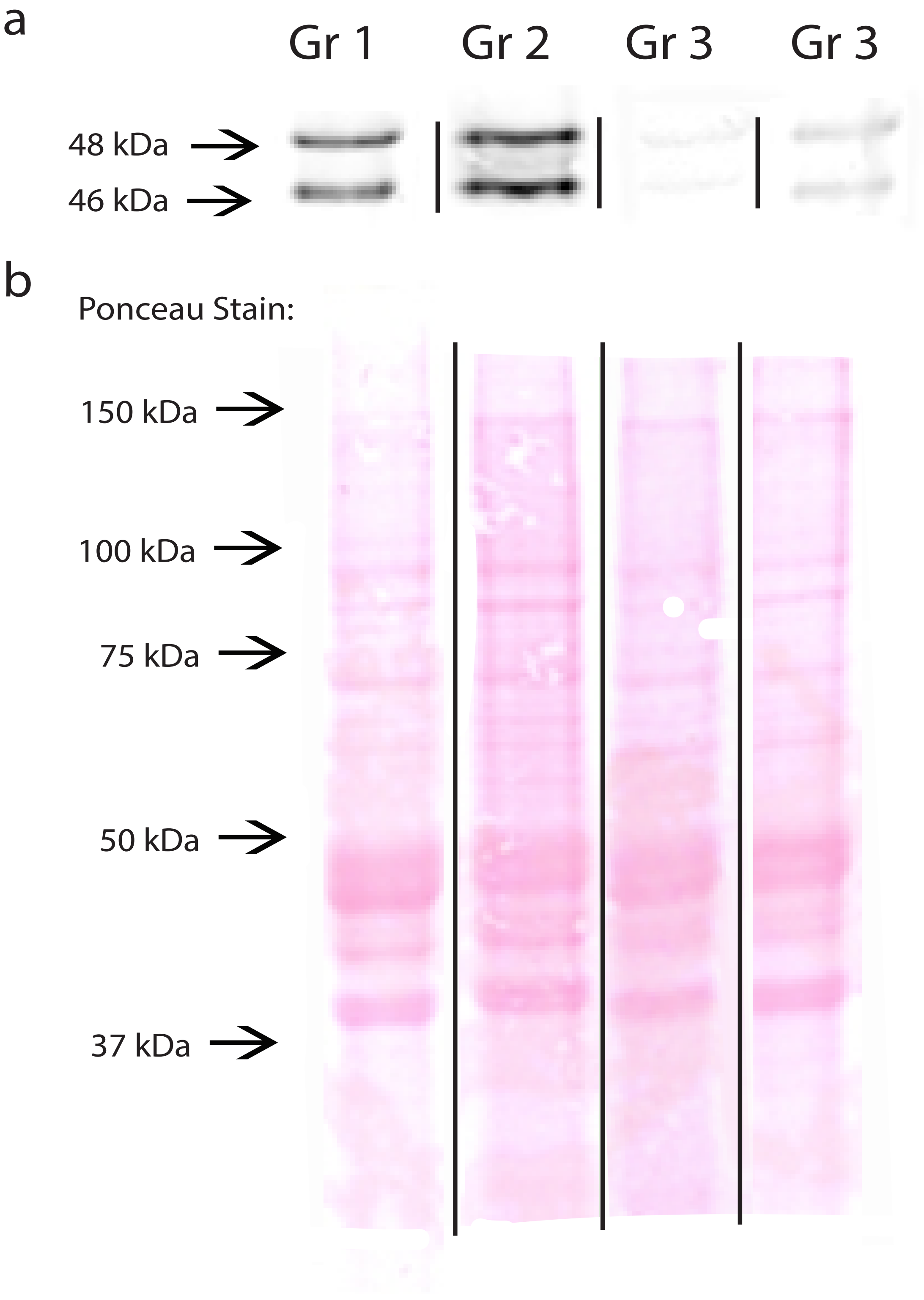

a
Gr 1
Gr 2
Gr 3
Gr 3
48 kDa
46 kDa
b
Ponceau Stain:
150 kDa
100 kDa
75 kDa
50 kDa
37 kDa

Fig. S3

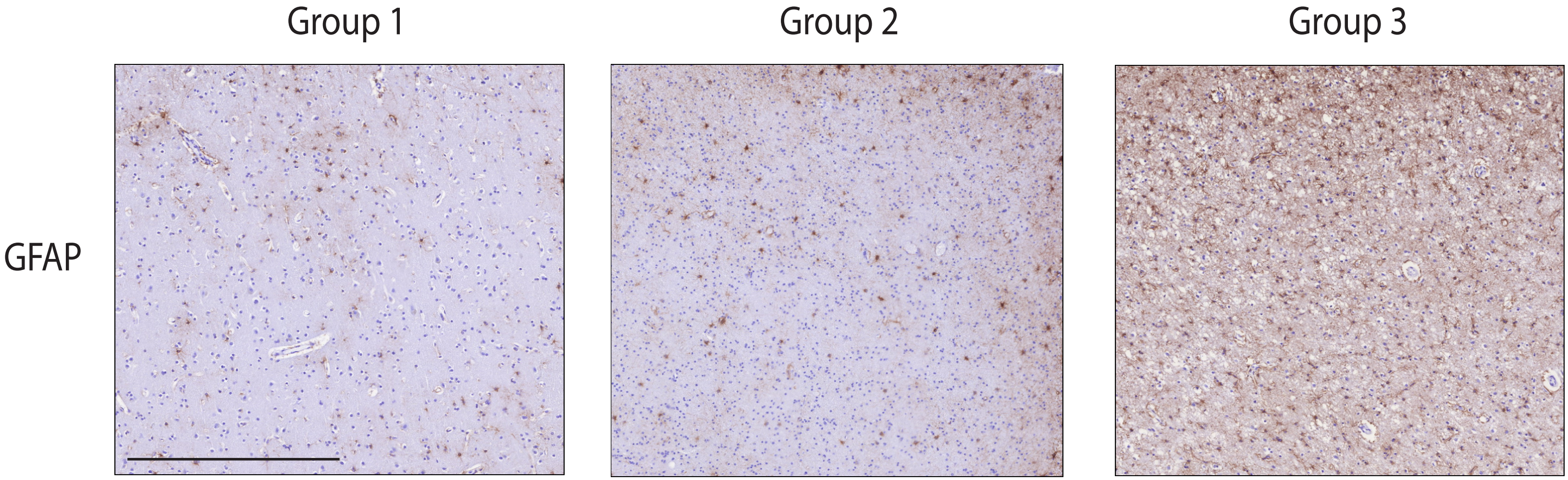

Fig. S4

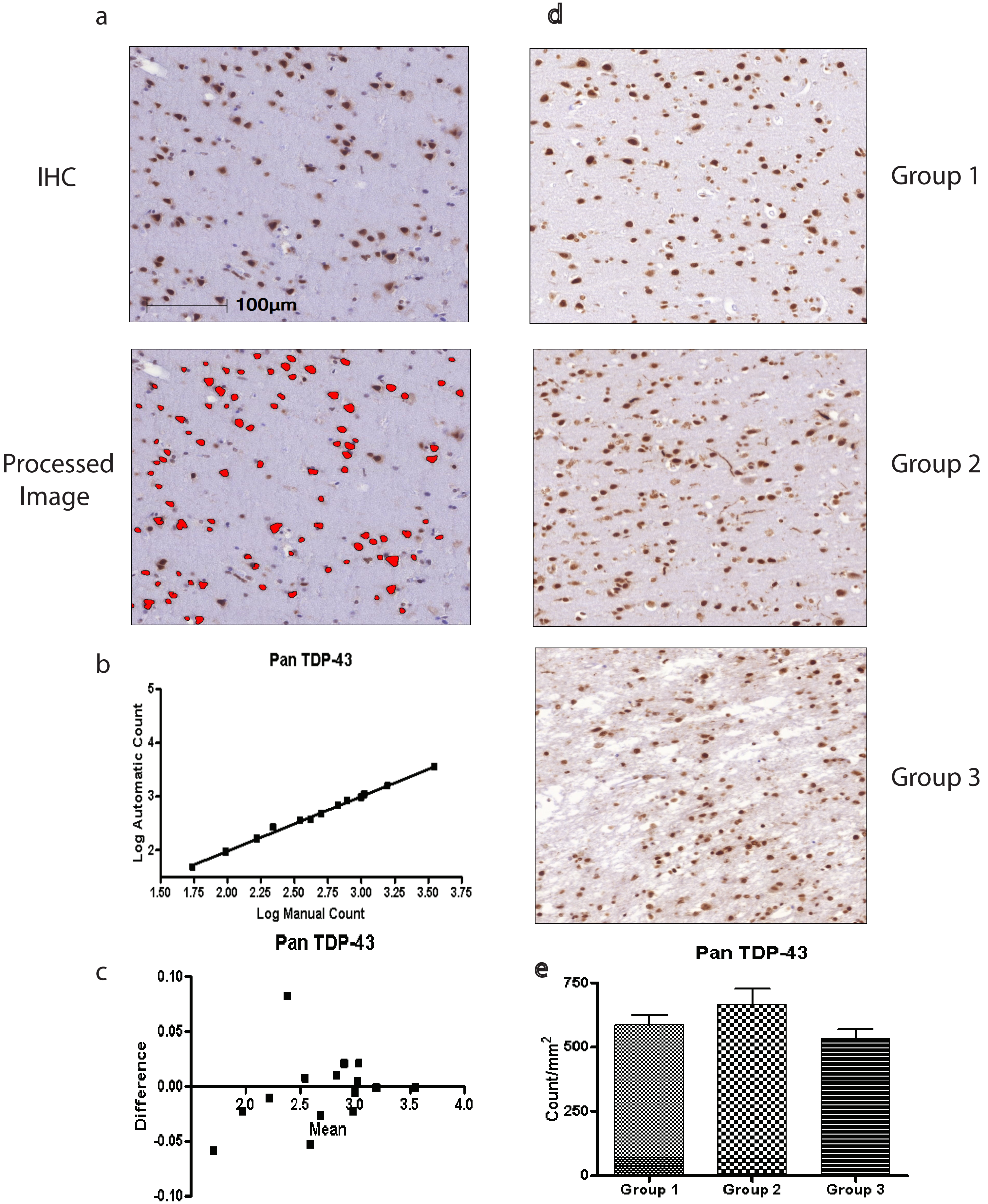

a
IHC
100µm
Processed Image
b
Pan TDP-43
Log Automatic Count
Log Manual Count
c
Pan TDP-43
Difference
Mean
d
Group 1
Group 2
Group 3
e
Pan TDP-43
Count/mm²
Group 1
Group 2
Group 3

Table S1 Focused analyses of bvFTLD-TDP recapitulate spread of pathology and genetic differences.

Measure	Estimate	Standard Error	Chi-Squared with DF=1 (P-Value)	OR (95% CI)	Chi-squared (DF) (P-Value)
**Phase**					
I[1]	-2.057	0.658	9.78 (0.0018)	0.13 (0.04-0.46)	6.70 (2) (0.0352)
II[2]	-0.422	0.421	1.01 (0.3160)	0.66 (0.29-1.50)	
III/IV[3]	2.478	0.527	22.09 (<0.0001)	11.92 (4.24-33.52)	
**Mutation**					
Non-C9/GRN[4]	0.492	0.860	0.33 (0.5675)	1.64 (0.30-8.82)	5.45 (2) (0.0656)
C9orf72[5]	-1.721	0.664	6.72 (0.0095)	0.18 (0.05-0.66)	
GRN[6]	1.230	0.996	1.52 (0.2169)	3.42 (0.49-24.07)	

[1]Phase III/IV is reference group.
[2]I is reference group.
[3]II is reference group.
[4]*GRN* is reference group.
[5]Non-C9/GRN is reference group.
[6]*C9orf72* is the reference group.
*GEE using a proportional odds model for Group 1-2 based on 80 observations. I- orbital frontal cortex; II- mid-frontal, anterior cingulate, entorhinal and superior temporal cortices; III- motor, sensory, and angular cortices; IV- visual cortex. Regions III and IV are combined due to small number of observations in each region.

Table S2 Focused analyses of non-bvFTLD-TDP recapitulate spread of pathology and genetic differences.

Measure	Estimate	Standard Error	Chi-Squared with DF=1 (P-Value)	OR (95% CI)	Chi-squared (DF) (P-Value)
**Phase**					
I[1]	-0.863	0.347	6.18 (0.0129)	0.42 (0.21-0.83)	10.22 (2) (0.0060)
II[2]	-0.139	0.448	0.10 (0.7566)	0.87 (0.36-2.10)	
III/IV[3]	1.002	0.282	12.67 (0.0004)	2.72 (1.57-4.73)	
**Mutation**					
Non-C9/GRN[4]	1.143	0.551	4.30 (0.0380)	3.14 (1.07-9.23)	4.62 (2) (0.0991)
C9orf72[5]	-0.796	0.488	2.66 (0.1028)	0.45 (0.17-1.17)	
GRN[6]	-0.347	0.543	0.41 (0.5235)	0.71 (0.24-2.05)	

[1]Phase III/IV is reference group.
[2]I is reference group.
[3]II is reference group.
[4]*GRN* is reference group.
[5]Non-C9/GRN is reference group.
[6]*C9orf72* is the reference group.
*GEE using a proportional odds model for Group 1-2 based on 193 observations. I- orbital frontal cortex; II- mid-frontal, anterior cingulate, entorhinal and superior temporal cortices; III- motor, sensory, and angular cortices; IV- visual cortex. Regions III and IV are combined due to small number of observations in each region.

Table S3 Superior temporal cortex tissue recapitulates genetic differences in FTLD-TDP.

Mutation Status	Count<br>Total %<br>Col %<br>Row %	Group 1	Group 2	Group 3	Total
	Non-C9/GRN	11<br>19.3%<br>64.7%<br>39.3%	12<br>21.1%<br>66.7%<br>42.9%	5<br>8.8%<br>22.7%<br>17.9%	28<br>49.1%
	C9orf72	2<br>3.5%<br>11.8%<br>11.8%	4<br>7.0%<br>22.2%<br>23.5%	11<br>19.3%<br>50.0%<br>64.7%	17<br>29.8%
	GRN	4<br>7.0%<br>23.5%<br>33.3%	2<br>3.5%<br>11.1%<br>16.7%	6<br>10.5%<br>27.3%<br>50.0%	12<br>21.1%
	Total	17<br>29.8%	18<br>31.6%	22<br>38.6%	57

*A 2-sided Fisher's exact test finds a significantly different distribution of Groups in the three mutation statuses (p=0.018).
